# Mauriac Syndrome in Sudanese Children: An Old Syndrome Still Existing in Resource-Limited Countries

**DOI:** 10.1155/pedi/7047312

**Published:** 2025-08-26

**Authors:** Mariam M. Ismail, Olivia A. Al-Hassan, Ghassan Mohamadsalih, Mohamed A. Abdullah

**Affiliations:** ^1^Pediatric Endocrinology Department, Gaafar Ibn Auf Pediatric Tertiary Hospital, Khartoum, Sudan; ^2^Endocrine and Diabetes Unit, Aladan Hospital, Hadiya, Kuwait; ^3^Division of Endocrinology, Department of Pediatric Medicine, Sidra Medicine, Doha, Qatar; ^4^Department of Pediatrics and Child Health, Faculty of Medicine, University of Khartoum, Khartoum, Sudan

## Abstract

**Objective:** Mauriac syndrome (MS) is a rare condition linked to inadequate glycemic control in type 1 diabetes mellitus (T1DM) and has also rarely been reported in patients with neonatal diabetes. MS manifests as growth failure, delayed puberty, cushingoid features, and hepatomegaly. The condition can be associated with complications like dyslipidemia, retinopathy, and nephropathy. The main objective of this study was to describe the magnitude of the condition, clinical features, management, and outcome of Sudanese children and adolescents with MS due to inadequate control of diabetes in our center.

**Study Design and Methods:** This is a cross-sectional hospital-based study. All medical records of patients with MS were reviewed. Data, including demographics, clinical features, investigations, management, and outcome, were obtained. Patients were re-educated and management intensified then followed up.

**Results:** Thirty-seven MS patients were enrolled in this study, with a male predominance of 59.5%. Neonatal diabetes was diagnosed in 5.4% of the patients, while others had T1DM. The median age at diagnosis of MS was 12 years. The diagnosis was based solely on clinical findings, including a history of prolonged unsatisfactory glycemic control, short stature, and hepatomegaly. Regarding the outcome, eight children (21.6%) were lost to follow-up, one patient died (2.7%), seven (18.9%) had a static condition, and those who showed improvement were 21 (56.8%). Signs of improvement were a decrease in liver size with or without an increase in growth velocity. Nephropathy was the most common associated complication; it was seen in 33.3% of our cohort. Some got it at a very young age.

**Conclusions:** Despite many efforts that have been made to achieve better glycemic control in children with T1DM, MS still exists in our setting. Though liver biopsy is the gold standard for diagnosis, being invasive, the diagnosis could be made conservatively, based on clinical features and response to treatment. The condition can be reversed with good metabolic control.

## 1. Introduction

Mauriac syndrome (MS) is a rare condition associated with poorly controlled type 1 diabetes mellitus (T1DM), resulting in growth failure, delayed puberty, hepatomegaly, and cushingoid features [1, 2]. It is also reported in patients with neonatal diabetes [3]. Patients with MS can exhibit either the obese or the nonobese variety with other features of poorly controlled diabetes, such as hepatomegaly, limited joint mobility, tight waxy skin, moon faces, protuberant abdomen, and proximal muscle wasting [4]. The condition can be associated with complications like dyslipidemia, retinopathy, and nephropathy [5]. Most reported cases were in children and adolescents with an equal sex distribution [6].

It was first described in 1930 by Pierre Mauriac [7]. Since then, it has been described in many case reports. Fitzpatrick et al. [4] documented 31 patients with MS in a retrospective study with a median age of 15.1 years (IQR 14–16.2).

MS is considered to be a benign and reversible condition with good outcomes [8]. Hepatomegaly in MS is due to glycogen accumulation rather than fatty infiltration, and different terms are used, such as liver glycogen storage, liver/hepatic glycogenosis, and recently glycogenic hepatopathy (GH) [7, 9]. The pathophysiology is not well understood, but it is thought to be the sequel of fluctuations in glucose levels with hyperglycemia, hypoglycemia, and hyperinsulinization [7]. It is believed that hyperglycemia and the consequent large amount of daily insulin dose per weight promote hepatic glycogen deposition, leading to liver damage and the release of aminotransferases [10, 11]. Recently, MacDonald et al. [12] discovered a heterozygous mutation found in a subunit of the liver glycogen phosphorylase kinase enzyme, which catalyzes the first step in glycogen breakdown in the liver. He proposed that this mutation could be related to the patient's predisposition to develop MS [12, 13]. In GH, there are abnormally elevated cortisol levels, which lead to cushingoid features, delayed puberty, secondary amenorrhea, and fatty acid mobilization, giving rise to dyslipidemia. These patients also have low insulin-like growth factor-1 (IGF-1) levels despite normal levels of growth hormone, resulting in growth failure [14]. The coexistence of some risk factors for GH, such as low socioeconomic status, adolescent age, and high daily insulin, is described by recent reports [9, 15, 16].

Radiological imaging studies like computed tomography and magnetic resonance imaging (MRI) are useful for diagnosing GH. However, their sensitivity and specificity are not established for this condition [15]. Ultrasound scan in GH shows hepatomegaly and hyperechogenic parenchyma that cannot be distinguished from nonalcoholic fatty liver disease (NAFLD) [17]. Liver biopsy is the gold standard GH diagnostic method, though it is invasive. The main GH histological features include pale swollen hepatocytes reflecting the marked glycogen accumulation, and no or mild fatty change, leading to minimal inflammation and spotty lobular necrosis. In addition, it usually shows intact architecture and no significant fibrosis [15]. A most recent systematic review stated that GH could be diagnosed based on clinical findings and the response to treatment, with deferring liver biopsy only for cases of doubtful diagnosis or poor clinical response [15].

The main pillar of MS management is to improve glycemic control by intensifying the insulin regime [1]. Despite the lack of data about vitamin D status in MS, several studies pointed out that vitamin D supplement has a positive impact on both HbA1c and metabolic status [18].

The main objective of this study was to obtain basic data, including clinical features and management and outcomes of Sudanese children and adolescents with MS. To the best of our knowledge, nothing has been recently published on this subject from Africa or the Middle East. While MS may be primarily seen in under-resourced settings, this is a syndrome that should be of interest to clinicians managing migrants and refugees from such regions.

## 2. Methods

### 2.1. Study Design and Population

This is a cross-sectional hospital-based study. All medical records of patients with MS who were seen in Gaafar Ibn Auf (GIA) Pediatric Tertiary Hospital and Sudan Childhood Diabetes Center (SCDC) from January 2006 to October 2022 were reviewed. Children and adolescents below the age of 18 years with T1DM and HbA1c above 7% (53 mmol/mol) were considered to have MS if they were short (height more than 2 SD below the mean for age and sex or height more than 1.5 SD below mid-parental height) with hepatomegaly, delayed puberty (lack of breast development by the age of 13 years in girls and no testicular enlargement at 14 years for boys). Patients with other chronic illnesses, autoimmune conditions, and significantly deficient data or loss to follow-up were excluded from the study.

Data, including demographics, clinical features, investigations, and management, were obtained. Patient's tribes were classified according to the three major African linguistic families in Sudan [19]. Bone age was assessed by using the Greulich and Pyle atlas. Liver size and echogenicity were evaluated by using ultrasound. The enzyme kinetic reaction method was used to assess aspartate aminotransferase (AST), alanine transaminase (ALT), and alkaline phosphatase (ALP). Liver enzymes were considered high if they were above normal ranges for our laboratory. Vitamin D status was assessed by measuring 25(OH)D3 using fluorescence immunoassay (FIA). Vitamin D level below 20 ng/mL was considered deficiency and a level of 21–29 ng/mL was insufficient, while levels of 30–100 ng/mL were sufficient [20]. Microalbuminuria was evaluated by using FIA, and considered positive if the albumin/creatinine ratio (ACR) was 2.5–25 mg/mmol in males and 3.5–25 mg/mmol in females, indicating diabetic nephropathy according to the International Society for Pediatric and Adolescent Diabetes (ISPAD) guidelines 2018 [21]. Lipid profile was assessed by using the endpoint reaction method. HbA1c% was evaluated by nephelometry and turbidity technique by using the semiautomated specific protein analyzer (MISPA-i2 analyzer). Luteinizing hormone (LH) and follicular-stimulating hormone (FSH) were measured using the enzyme-linked immunosorbent assay (ELISA) method. The normal range of LH for men is 1.24–7.8 IU/mL, and for women: the follicular phase of the menstrual cycle is 1.68–15 IU/mL and for midcycle peak is 21.9–56.6 IU/mL. FSH normal range: during puberty is 0.3–10 IU/L. Fundal examination through dilated pupils was performed for retinopathy evaluation by an ophthalmologist.

All the patients were re-educated and counseled about diet control and exercise. Patient's insulin regimen and doses were intensified by using basal-bolus regimen in six patients (16.2%). Thirty-one patients (83.7%) continued on twice-daily premixed insulin with the introduction of a fixed dose of regular insulin prelunch. On follow-up, the criteria of MS resolution were looked for, including a decrease in liver size with or without an increase in growth velocity.

Ethical approval was obtained from GIA and SCDC ethical approval boards. Verbal consent was taken from patients and their families.

### 2.2. Statistical Analysis

All the data were entered into the computer and analyzed by using a Statistical Package for the Social Science software, version 26. Descriptive variables were presented in frequency tables with percentages and graphs. Descriptive analysis was performed for all study variables with mean (standard deviation) or median (interquartile range) for quantitative data and frequencies with proportion for qualitative data. Bivariable analysis was used to determine the associations between the main outcome variable and the other relevant factors using the chi-square test to compare nonparametric data (categorical variables), and the *t*-test was used for quantitative variables as well. A *p*-value less than 0.05 was considered statistically significant.

## 3. Results

The demographic data and history are shown in Table 1. A total of 37 patients were enrolled in this study, with a male predominance of 22 (59.5%). Most of the patients had a duration of diabetes of less than 5 years. Two cases (5.4%), both of them males, had neonatal diabetes based on age at diagnosis. The molecular genetics of one of them at Exeter Clinical Laboratory International in the United Kingdom showed a homozygous mutation in the GCK gene exon 3.

### 3.1. Clinical Characteristics

The clinical findings are shown in Table 2. All the patients were short, had hepatomegaly, and all of those who were at the age of puberty (19 patients) had delayed puberty with hypogonadotropic hypogonadism. Delayed bone age was found in 90% of the patients. The total daily insulin dose was less than 0.5 IU/kg/day in one patient (2.7%), between 0.5 and 1 IU/kg/day in 20 patients (54.1%) and 16 patients (43.2%) required between 1 and 2 IU/kg/day. The enrolled patients were found to use either three daily injections of a mixed human insulin 17 (45.95%) (prebreakfast and dinner) and regular insulin (prelunch) regimen, or twice-daily injection of mixed human insulin 17 (45.95%) and basal-bolus regimen with insulin analogs in only 3 (8.1%) of the patients. Those with no means for self-home blood glucose monitoring were 25 (67.6%). The patients who frequently missed insulin doses were 30 patients (81.1%).

Thirty-one patients (83.8%) had repeated episodes of DKA less than 3 times/year versus 6 (16.2%) who had it at least 3 times/year. All patients had recurrent episodes of hypoglycemia as follows: 24 of the children (64.9%) had less than 5 times/year, nine patients (24.3%) had 5–10 times/year, and four patients (10.8%) had more than 10 times/year. Twenty-four (35.1%) of them had asymptomatic hypoglycemia. The majority of patients (86.5%) had more than 10% of HbA1c, just five patients (13.5%) had HbA1c between 7% and 10%, and no patients below 7%.

Most of the patients had normal ALT 60% and AST 74.2%. Liver ultrasound revealed an enlarged hyperechoic liver in 63.3% of the patients, while 36.7% showed an enlarged liver with normal echogenicity. More than three-quarters of the patients 77.8%, had insufficient vitamin D levels, while three patients 11.1% had normal and a similar number had vitamin D deficiency. High cholesterol levels were seen in seven cases (24.3%), while triglyceride and low-density lipoprotein (LDL) were high in 13.5% and 5.4%, respectively. The commonest chronic complications are shown in Table 2. Nephropathy was seen as early as 6 years of age in a girl who had poor control T1DM for 3 years, one of the patients of diabetic nephropathy developed end stage renal disease (ESRD) and underwent hemodialysis but unfortunately died.

Hypertension was seen in eight patients (21.6%). All cases of diabetic retinopathy had mild and moderate nonproliferative retinopathy.

### 3.2. Management Progression and Outcome

All the patients were re-educated, and their insulin regimen and doses were intensified. Nineteen patients (51.4%) were admitted to GIA hospital and 18 patients (48.6%) were managed in the outpatient clinic. A basal-bolus regimen was identified in six patients (16.2%), three of them used insulin analogs and one patient used NPH twice as a basal insulin and regular as preprandial. Thirty-one patients (83.7%) continued on twice-daily premixed insulin with the introduction of regular insulin prelunch. The mean insulin dose was increased to 1.5 IU/kg/day. This was in addition to the provision of meters and strips.

**Figure 1:**
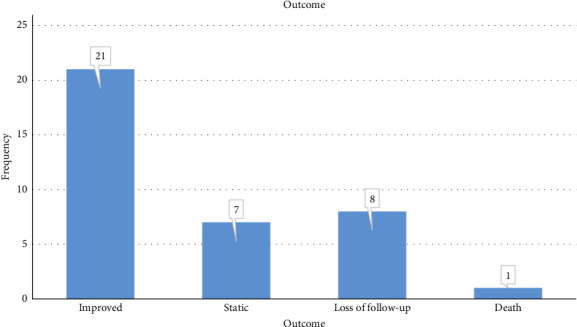
Outcome of management of the MS patients. One patient died due to ESRD and mucormycosis infection.

Figure 1: shows the outcome of MS patients

For those who showed signs of improvement; 54.6% of patients took more than 1 year from the initial presentation, while 13.6% needed 6 months to 1 year, a period of 3–6 months was seen in 27.2% and 4.5% needed less than 3 months.

**Figure 2:**
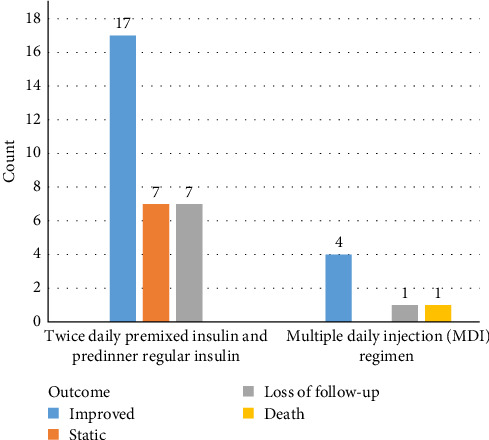
The number of patients and outcome of patients who took twice daily premixed insulin and predinner regular insulin and those who took MDI regimen.

The outcome of the patients who took twice daily premixed insulin and prelunch regular insulin and those who took the MDI regimen is shown in Figure 2.

The first sign of improvement was a decrease in liver size. The liver size returned to normal in 52.1%, reduction in size in 39.1% and 8.7% showed no change. About 55.6% of the cases still had a height more than 3 SD below the mean, and the rest remained between −2 and −3 SD and −1 and −2 SD in equal percentages of 22.2%. All children and adolescents in the pubertal age (19 patients) had delayed puberty with low LH and FSH. During follow-up six patients (31.6%) showed signs of puberty. All patients who had diabetic nephropathy received angiotensin-converting enzyme inhibitor (lisinopril) and had follow-up with a pediatric nephrologist. One of them developed ESRD and underwent hemodialysis but unfortunately died. The other patients had normal renal function. The two patients with diabetic retinopathy were referred to a pediatric ophthalmologist for follow-up, they were reported to have mild and moderate nonproliferative retinopathy and normal vision. Those with dyslipidemia were managed according to ISPAD guidelines by improving glycemic control, dietary changes and increased exercise. No sufficient follow-up data regarding lipid profile was documented in the records. All patients with insufficient and deficient vitamin D received vitamin D therapy.

HbA1c was still high in the majority; (58.8%) had HbA1c more than 10% (˃86 mmol/mol), and it dropped to 7%–10% (61–86 mmol/mol) in just 32.2%, and to less than 7% (61 mmol/mol) in only 5.9%. There was a significant drop in HbA1c in the group who showed clinical improvement compared to those these who did not show improvement (*p*-value 0.048).

## 4. Discussion

Even though MS has been reported previously from Sudan and Nigeria [22, 23] to the best of our knowledge, this is the first paper from Africa with an attempt to describe the characteristics of patients with MS in such a large number. Despite the serious efforts that have been made to achieve better glycaemic control in children with T1DM, MS cases are still seen from time to time, particularly in limited resources areas where management of DM is hectic. Although this syndrome is rare in developed countries, it should be of interest to clinicians managing migrants and refugees from such regions.

Male predominance was observed in this study 59.5%. This is contrary to what was observed in many reported cases in children and adolescents as sex distribution was equal [5, 6]. The median age of diagnosis of MS was 12 years, which is similar to what was observed in other reports [5]. Most of the patients had a duration of diabetes of less than 5 years, and the median age of diagnosis of diabetes was 6 years. This differs from what was mentioned by Fitzpatrick et al. [4] where the median age of diagnosis of diabetes was 10 years.

Two male cases were diagnosed with neonatal diabetes, one of them had a homozygous mutation in the GCK gene. This is comparable to what was described by Chai-udom et al. [3] who reported a presentation of MS in a 9-year-old girl who was diagnosed with neonatal diabetes due to KCNJ11, indicating that MS is not exclusive to T1DM patients.

The bulk of the patients 51.4% were referred from various states of Sudan to our center, thus representing different parts of Sudan and ethnic groups. Patients who belonged to the Afro–Asiatic group were 64.6% and those who belonged to Niger–Congo represented 13.5%, which may indicate a genetic predisposition to MS. Recently, MacDonald et al. [12] discovered a heterozygous mutation found in a subunit of the liver glycogen phosphorylase kinase enzyme which catalyzes the first step in glycogen breakdown in the liver. He proposed that this mutation could be related to the patient's predisposition to develop MS [12, 13]. However, further genetic studies with larger numbers are needed.

Regarding the associated complications, the commonest was nephropathy 33.3% which was seen in as early as 6 years of age in a girl who had poor control of T1DM for 3 years. She presented with hypertension and albuminuria, and this has never been mentioned in the literature at such an early age. Children with GH and those with out of target in range (TIR) diabetes control should be screened for nephropathy at a younger age than what is recommended by ISPAD. Retinopathy was seen in 12.5% of the cases. The youngest patient with retinopathy was a girl of 11 years. Dyslipidemia was detected in (25.9%) and has been described in many case reports [14]. Although improvement of dyslipidemia is less commonly reported, early screening is important to prevent cardiovascular complications [14].

In contrast to what was observed in other studies [6, 24], in which elevated transaminases were documented and reversed after glycemic control, most of our patients had normal liver enzymes similar to some reports where there were no liver derangements [12, 25]. There are several differential diagnoses for patients with DM, hepatomegaly, and elevated transaminase levels, which include: GH, which is most likely in patients with T1DM, NAFLD, mainly in T2DM patients, celiac disease, autoimmune hepatitis, viral hepatitis, hemochromatosis, and Wilson's disease [7, 26]. We did not screen our cases for these.

Liver ultrasound revealed an enlarged hyperechoic liver in 63.3% of our patients, which is consistent with findings reported by Mertens et al. [17]. It is important to note that distinguishing between GH and NAFLD using sonography is not feasible without a liver biopsy. MRI can aid in differentiating GH [15]. However, MRI is not easily accessible or affordable in most resource-limited countries.

Despite this, the clinical and biochemical features align with a diagnosis of GH [17]. We did not conduct any liver biopsies, as they are invasive procedures, nor did we utilize MRI in any of our cases due to financial constraints.

In this study, we noticed that HbA1c was more than 10% in 86.5% of the patients, was more prominent in adolescent age and correlated with poor compliance, recurrent DKA and hypoglycemia, low socioeconomic status, and illiteracy. This is in agreement with what was mentioned by many authors [1, 5, 25]. Poverty is the primary reason for inadequate diabetes management. The cost of diabetes management is high, and most patients come from low socioeconomic backgrounds. Only 8.1% of the patients used a basal-bolus insulin regimen, primarily because long-acting insulins are often unavailable in many areas and can be prohibitively expensive, as patients have to pay out of pocket. However, some basal insulins are now provided free of charge by the Sudan Childhood Diabetes Association (SCDA). NPH insulin can be administered twice a day as a basal option, combined with regular human insulin for premeal dosing. We have used this approach in a limited number of our patients. The average insulin dose in 43.2% of the patients was 1–2 IU/kg/day. This aligns with the pathophysiology of GH as it is thought to be the sequel of fluctuations in glucose level and hyperinsulinization [7]. In this study, a very limited number of patients were put on intensive therapy due to lack of facilities. More studies using intensive therapy including MDI and pumps are needed to see their effect on the outcome of MS. In addition to intensifying insulin therapy, control of diabetes needs facilities for self-monitoring of blood glucose and urine testing strips for ketones. Most of the patients were not monitoring their blood glucose at home because of unavailability. However, these are now provided free by SCDA, an NGO which is supported by international NGOs like Life for a Child (LFAC) program and Changing Diabetes in Children (CDIC). SCDA has now established clinics in all the states of Sudan. Also, studies on monitoring using continuous subcutaneous glucose monitoring (CGM) and seeing patterns of TIR are suggested as TIR is more reliable than HbA1c in cases with fluctuating hyper and hypoglycemia.

Achievement of metabolic control results in an absolute remission of clinical and laboratory abnormalities, which can be as quick as in days to weeks [1]. Hepatomegaly and elevated liver enzymes are among the first issues to improve, while acceleration in growth varies and may take a longer time [25]. In this study, enhancing management through education and optimizing the insulin regimen resulted in a reduction of liver size in the majority of patients; only 8.7% showed no response. The possible explanation that the stature of 55.6% remained more than 3 SD below the mean, can be due to the longer time needed to achieve growth improvement. Perhaps the follow-up duration of 1 year or less in our study was too short to see improvement in growth. This is similar to what was observed by Lombardo et al. [15]. Growth in patients with DM is still less than expected. The cause of poor growth in such patients is multifactorial [12]. In MS, delayed growth and puberty are caused by inadequate tissue glucose, decreased IGF-1 levels, receptor impairment or resistance to growth hormone action, and hypercortisolism, which is the cause of cushingoid features in this condition [2, 27]. However, most of our cases had no cushingoid body habitus and we did not measure cortisol level. In our cases with delayed puberty, there was hypogonadotropic hypogonadism and if after controlling diabetes it persists possibly these cases may benefit from induction of puberty by sex hormones as in the case of constitutional delay or hypogonadotropic hypogonadism. This issue needs further studies.

Vitamin D supplements can have a positive impact on both HbA1c and metabolic status as part of the intensified management of DM [17]. In this series, the majority of patients, though living in a sunny country, had insufficient vitamin D levels, and thus patients were supplemented with vitamin D. All of them had negative celiac screening.

## 5. Conclusion

Despite many efforts that have been made to achieve better glycemic control in children with T1DM, MS still exists, particularly in resource limited settings such as Sudan as where the management of DM is costly. Although liver biopsy is the gold standard for the diagnosis of MS, it is invasive, and thus a diagnosis can be made based on clinical grounds and management response. The condition is preventable and reversible with good metabolic control. More studies are needed to intensify and control management, including using current technology to prevent and control MS.

The main limitations of this study were that it was a retrospective study, patients were not followed up long enough to see the long-term outcome and a very limited number were put on intensive therapy.

## Figures and Tables

**Table 1:** Patient's demographic data and history.

Variables	Overall *n* = 37 Median, interquartile
Age at diagnosis of MS (years)	12 (6–17)
Age at diagnosis of diabetes (years)	6 (0.08–16)
Residency inside Khartoum	18 (48.6%)
Residency outside Khartoum	19 (51.4%)
Afro-Asiatic group	64.6%
Nilo-Saharan group	24.3%
Niger-Congo group	13.5%
Low educational achievement of the parents	23 (62.2%)
Low socioeconomic status	33 89.2%)
DKA > 3 episodes/year	31 (83.8%)
Hypoglycemia	37 (100%)
Poor dietary control	35 (94.6%)

**Table 2:** Patient's clinical findings:.

Variables	Overall *n* = 37 Median and interquartile
Weight SDS	−3(−9.10–−1)
Height SDS	−4(−7.8–−2)
BMI SDS	−2(−4–−1)
Short stature	37 (100%)
Hepatomegaly	37 (100%)
Delayed puberty	19 (100%)
Jaundice	23 (62.2)
Cushingoid body habitus	4 (10.8%)
Limited joints mobility	20 (54.1%)
Nephropathy	11 (33.3%)
Dyslipidemia	7 (25.9%)
Retinopathy	2 (12.5%)

## Data Availability

The quantitive data used to support the findings of this study are available from the corresponding author upon reasonable request.

## Nomenclature

MS: Mauriac syndrome
T1DM: Type 1 diabetes mellitus
GH: Glycogenic hepatopathy
FIA: Fluorescence immunoassay
AST: Aspartate aminotransferase
ALT: Alanine transaminase
ALP: Alkaline phosphatase
DKA: Diabetes ketoacidosis
LDLs: Low-density lipoproteins
ESRD: End-stage renal disease
MRI: Magnetic resonance imaging
IGF-1: Insulin-like growth factor-1.

## References

[1] Suryono S., Malik M. U., Yung C. K., Chyuan E. L. C., Metussin P. A. P. (2021). Case Report of a Patient With Mauriac Syndrome. *Asian Journal of Case Reports in Medicine and Health*.

[2] Rai S., Kaur A., Kaul V., Meena L., Metha S. (2017). Mauriac Syndrome: Rare Complication in Type-1 Diabetic Children. *Pediatrics & Therapeutics*.

[3] Chai-udom R., Sahakitrungruang T., Wacharasindhu S., Supornsilchai V. (2016). A Girl With Permanent Neonatal Diabetes due to KCNJ11 Mutation Presented With Mauriac Syndrome After Improper Adjustment in Sulfonylurea Dosage Over 6 Years. *Journal of Pediatric Endocrinology and Metabolism*.

[4] Fitzpatrick E., Cotoi C., Quaglia A., Sakellariou S., Ford-Adams M. E., Hadzic N. (2014). Hepatopathy of Mauriac Syndrome: A Retrospective Review From a Tertiary Liver Centre. *Archives of Disease in Childhood*.

[5] Thakkar U. G., Vanikar A. V., Trivedi H. L. (2017). Revisit of a Rare Complication of Type 1 Diabetes Mellitus: Mauriac Syndrome. *Practical Diabetes*.

[6] Ahajjaj A. H., Aljishi F. K. (2021). Mauriac Syndrome Still Exists in Poorly Controlled Type 1 Diabetes: A Report of Two Cases and Literature Review. *Cureus*.

[7] Sherigar J. M., Castro J. D., Yin Y. M., Guss D., Mohanty S. R. (2018). Glycogenic Hepatopathy: A Narrative Review. *World Journal of Hepatology*.

[8] Sarkhy A. A. A., Zaidi Z. A., Babiker A. M. (2017). Glycogenic Hepatopathy, an Underdiagnosed Cause of Relapsing Hepatitis in Uncontrolled Type 1 Diabetes Mellitus. *Saudi Medical Journal*.

[9] Khoury J., Zohar Y., Shehadeh N., Saadi T. (2018). Glycogenic Hepatopathy. *Hepatobiliary & Pancreatic Diseases International*.

[10] Otto-Buczkowska E., Jainta N. (2017). Mauriac Syndrome —is Already a History?. *Clinical Diabetology*.

[11] Touilloux B., Lu H., Campos-Xavier B. (2021). Elevated Lactate in Mauriac Syndrome: Still a mystery. *BMC Endocrine Disorders*.

[12] MacDonald M. J., Hasan N. M., Ansari I. H., Longacre M. J., Kendrick M. A., Stoker S. W. (2016). Discovery of a Genetic Metabolic Cause for Mauriac Syndrome in Type 1 Diabetes. *Diabetes*.

[13] Mitchell D. M. (2017). Growth in Patients With Type 1 Diabetes. *Current Opinion in Endocrinology, Diabetes & Obesity*.

[14] Fox M. T., Tamaroff J., Percy A. G. (2021). Hepatomegaly and Short Stature in a 14-Year-Old With Type 1 Diabetes Mellitus: Case Report. *Family Practice*.

[15] Lombardo F., Passanisi S., Gasbarro A., Tuccari G., Ieni A., Salnzao G. (2019). Hepatomegaly and Type 1 Diabetes: A Clinical Case of Mauriac’s Syndrome. *Italian Journal of Pediatrics*.

[16] Yadav J., Kumar R., Gupta S., Gupta A., Yadav A., Dayal D. (2021). Mauriac Syndrome: A Failure of Parent or Physician. *Tropical Doctor*.

[17] Mertens J., De Block C., Spinhoven M., Driessen A., Francque S. M., Kwanten W. J. (2021). Hepatopathy Associated With Type 1 Diabetes: Distinguishing Non-alcoholic Fatty Liver Disease From Glycogenic Hepatopathy. *Frontiers in Pharmacology*.

[18] Dehkordi E. H., Dehkordi V. H., Fatemi S. M., Zolfaghari M. (2018). Effect of Vitamin D Supplement Therapy on HbA1c and IGF-1 Levels in Children With Type 1 Diabetes Mellitus and Vitamin D Deficiency. *Electronic Journal of General Medicine*.

[19] Hassan H. Y., Underhill P. A., Cavalli-Sforza L. L., Ibrahim M. E. (2008). Y-Chromosome Variation Among Sudanese: Restricted Gene Flow, Concordance With Language, Geography and History. *American Journal of Physical Anthropology*.

[20] Holick M. F., Binkley N. C., Bischoff-Ferrari H. A., Gordon C. M., Heaney Hanley D. A., etal R. P. (2011). Evaluation, Treatment, and Prevention of Vitamin D Deficiency: An Endocrine Society Clinical Practice Guideline. *The Journal of Clinical Endocrinology & Metabolism*.

[21] -ISPAD clinical Practice Consensus Guidelines (2018). Microvascular and Macrovascular Complications in Children and Adolescents.

[22] Oyenusi E. E., Ezeani I. U. (2021). Mauriac Syndrome in a Nigerian Child With Type 1 Diabetes Mellitus: A Case Report. *Annals of Health Research*.

[23] Ilham Mohammed O. (2017). Mauriac Syndrome: A Diagnosis That Still Exists. *Academic Journal of Pediatrics and Neonatology*.

[24] Negm S., Elhariri A., Elhariri S. (2023). Mauriac Syndrome With Reversible Clinical and Laboratory Findings After Glycemic Control(Case Series). *Annals of Clinical and Medical Case Reports & Reviews*.

[25] Kocova M., Milenkova L. (2018). Old Syndrome-New Approach: Mauriac Syndrome Treated With Continuous Insulin Delivery. *SAGE Open Medical Case Reports*.

[26] Dolip W., Bourmanne E., Van Homwegen C., Van Nuffelen M. (2022). Persistent Hyperlactatemia in Decompensated Type I Diabetes With Hepatic Glycogenosis and Hepatomegaly: Mauriac Syndrome: A Case Report. *Journal of Medical Case Reports*.

[27] Elder C. J., Natarajan A. (2010). Mauriac Syndrome—A Modern Reality. *Journal of Pediatric Endocrinology and Metabolism*.

